# Temporal trends in long-term relative survival of cutaneous verrucous carcinoma: a period analysis from a large-scale SEER-based cohort

**DOI:** 10.3389/fmed.2026.1827764

**Published:** 2026-08-03

**Authors:** Siomui Chong, Congying Qiu, Santosh Chokkakula, Yu-Yen Yang, Liying Huang, Yuyi Ou, Xiaoxi Zhang, Tingting Lau, Ying Zhou, Rajeswari Chappa, Chengliang Yin, Jun Lyu, Liehua Deng

**Affiliations:** 1 Department of Dermatology, The First Affiliated Hospital of Jinan University and Jinan University Institute of Dermatology, Guangzhou, China; 2 Department of Dermatology, The University of Hong Kong-Shenzhen Hospital, Shenzhen, China; 3 Key Laboratory of Regenerative Medicine, Ministry of Education, Department of Developmental and Regenerative Biology, Jinan University, Guangzhou, China; 4 Department of Microbiology, Chungbuk National University College of Medicine and Medical Research Institute, Cheongju, Chungbuk, Republic of Korea; 5 Dr. Pong Dermatologic and Aesthetic Clinic, Taipei, Taiwan; 6 Clinical Research Center, The First Affiliated Hospital of Jinan University, Guangzhou, Guangdong, China; 7 Department of Gynaecology, Foshan Women and Children Hospital Affiliated to Guangdong Medical University, Foshan, Guangdong, China; 8 The First Affiliated Hospital of Jinan University, Guangzhou, China; 9 Bodhan Soft Technologies, Visakhapatnam, India; 10 Department of Biomedical Engineering, Faculty of Engineering, Universiti Malaya, Kuala Lumpur, Malaysia; 11 Department of Pharmacy, The First Affiliated Hospital of Jinan University, Guangzhou, Guangdong, China; 12 Department of Dermatology, The Fifth Affiliated Hospital of Jinan University, Heyuan, China

**Keywords:** cutaneous verrucous carcinoma, period analysis, racial disparities, relative survival, SEER database, socioeconomic status

## Abstract

**Background:**

Cutaneous verrucous carcinoma (CVC) is a rare, well-differentiated variant of squamous cell carcinoma characterized by slow progression, local invasiveness, and limited propensity for metastasis. Despite these clinical features, comprehensive population-based evidence on long-term survival outcomes is lacking. This study aimed to evaluate the long-term relative survival (RS) of CVC patients over 20 years using period analysis, providing insights to guide future prevention and therapeutic approaches.

**Methods:**

A retrospective cohort analysis was performed using the SEER 17 database (2000–2019) from the U.S. National Cancer Institute. All patients with histologically confirmed CVC were identified using ICD-O-3 histology code 8051/3 and primary site codes restricted to skin (C44.0–C44.9). Period analysis, a statistical approach that provides up-to-date survival estimates by incorporating recent follow-up data, was employed to calculate 3- and 5-year relative survival rates. Survival estimates were stratified by variables including age, sex, race, and socioeconomic status. Additionally, model-based projections for the 2020–2024 period were generated using an established generalized linear model to forecast future survival trends.

**Results:**

A total of 2,031 patients were included. Survival trends were heterogeneous across subgroups. In the most recent period (2015–2019), the overall 3- and 5-year RS rates were 85.3 and 82.9%, respectively. Male patients demonstrated improving RS over time, with 5-year RS reaching 87.6% in 2015–2019, while female patients exhibited a declining trend, with 5-year RS of 74.9% in the same period. Analysis of race revealed that White patients exhibited the highest 5-year RS at 84.8%, while Black patients had the lowest at 65.9%. Regarding the residential area, rural patients achieved a higher 5-year RS (87.4%) compared to metropolitan patients (81.9%) in 2015–2019. Socioeconomic factors also influenced outcomes, with patients in higher income brackets projected to achieve superior RS through 2020–2024. Although disparities in survival by sex, race, age, and socioeconomic status remained evident, model-based projections suggest continued divergence across subgroups through 2020–2024.

**Conclusion:**

Survival trends among CVC patients from 2000 to 2019 were heterogeneous across subgroups, with improving outcomes in males and younger patients but declining trends in females, older age groups, and urban residents. Persistent disparities by sex, race, income, and residence remain a significant concern. Targeted strategies are needed to address these inequities and improve outcomes for vulnerable groups.

## 1 Introduction

Cutaneous verrucous carcinoma (CVC) is a rare, well-differentiated, low-grade variant of squamous cell carcinoma (SCC) arising from the skin, characterized by slow progression, local invasiveness, and a notably low propensity for metastasis (1). It must be distinguished from verrucous carcinoma arising at non-cutaneous sites, including the oral cavity, larynx, and anogenital tract, which have distinct epidemiologies, risk factors, and clinical behaviors. The present study focuses exclusively on cutaneous primaries. Squamous cell carcinoma broadly accounts for approximately 20–50% of all skin cancers, with CVC representing a rare subtype within this category (2). Skin cancer represents approximately one-third of all newly diagnosed cancers worldwide, with non-melanoma skin cancers, including SCC and its variants, exhibiting a steadily increasing global incidence in recent years (3, 4). Unlike conventional SCC, CVC less frequently arises in sun-exposed areas, suggesting distinct pathogenic mechanisms and risk profiles (5). Patients with CVC typically experience a prolonged disease course, ranging from 1 to 8 years before diagnosis, with a median duration of 5 years. The tumor predominantly affects the skin of the soles, lower extremities, and other cutaneous surfaces (6).

Verrucous carcinoma can arise at multiple anatomical sites, and its epidemiology and risk factors vary accordingly. Penile verrucous carcinoma comprises approximately 5 to 24% of all verrucous carcinoma cases, with an estimated incidence ranging from 0.75–3 per million individuals (7, 8). At mucosal sites such as the oral cavity and larynx, risk factors including tobacco use, alcohol consumption, betel nut chewing, and human papillomavirus (HPV) infection have been consistently identified (9, 10). For cutaneous CVC specifically, chronic inflammation, immunosuppression, and prolonged mechanical irritation are among the implicated contributing factors. In contrast, the role of UV exposure remains less well-defined than in conventional cSCC (5). It is important to note that these site-specific risk factors are not universally applicable across all forms of verrucous carcinoma, and caution should be exercised when extrapolating etiological data between cutaneous and non-cutaneous subtypes. In light of the increasing global burden of non-melanoma skin cancers, a comprehensive understanding of the epidemiology and risk profile of CVC is critical for optimizing disease management strategies. Furthermore, the economic impact of non-melanoma skin cancers is substantial, encompassing both direct healthcare expenditures and indirect costs associated with productivity loss (11).

Cancer survival rates are fundamental indicators for assessing the effectiveness of cancer control strategies at both the regional and national levels. These metrics are typically obtained from clinical follow-up data, hospital-based registries, or, most robustly, from population-based cancer registries, which provide the most comprehensive and generalizable survival estimates for informing public health policy (12). Among the key survival measures are the observed survival rate (OSR), expected survival rate (ESR), and relative survival rate (RSR). Of these, RSR is considered the gold standard, as it accounts for competing causes of death and provides a more accurate reflection of cancer-specific net survival (13, 14). However, conventional survival estimates are often limited by a significant time lag due to the necessity for prolonged follow-up periods. In contrast, the 5-year RSR delivers more current and clinically meaningful data, offering timely insights into the effectiveness of contemporary treatment approaches (15).

To address the limitations inherent in conventional survival analysis methods, Brenner and Hakulinen introduced a model-based period analysis for predicting long-term cancer survival rates (16). This approach leverages sequential survival data such as from 2000–2004, 2005–2009, 2010–2014, and 2015–2019 to generate projections of 5-year survival rates for subsequent periods, including 2020–2024. Compared to traditional cohort-based methods, model-based period analysis provides more up-to-date and precise survival estimates by incorporating recent follow-up data and allowing for adjustment of key covariates such as sex, age, and tumor stage. While there is currently no universally standardized multivariate approach for analyzing relative survival rates, most existing studies rely on stratification by subgroups and calculation of RSRs within each category. Model-based period analysis, however, offers the added advantage of adjusting for confounding variables and enables formal hypothesis testing, making it a robust and valuable complement to traditional stratified methods (17).

Although CVC is generally associated with a favorable prognosis, reported survival rates vary considerably depending on patient characteristics, tumor stage, and anatomical site of origin (18, 19). Comprehensive population-based survival analyses utilizing period methods for cutaneous CVC specifically remain limited. In response to the increasing global cancer burden, model-based period analysis has been widely adopted in Western countries to monitor and project cancer survival trends (16, 17). This analytical approach has recently gained prominence in survival research, enabling more accurate projections of future 5-year survival outcomes. Thus, in the present study, we employed model-based period analysis to assess long-term survival trends among CVC patients from 2000 to 2019 using data from the Surveillance, Epidemiology, and End Results (SEER) database. Understanding these temporal trends is essential to inform future clinical practice and public health strategies targeting CVC.

## 2 Patient and methods

### 2.1 Data source

This retrospective cohort study was conducted using data from the SEER program, a comprehensive, population-based cancer registry sponsored by the U.S. National Cancer Institute. Specifically, data were extracted from the “Incidence SEER Research Plus Data, 17 Registries, 2000–2019” dataset, which covers approximately 26% of the U.S. population (20, 21). The SEER database is widely recognized as a gold standard for cancer surveillance, offering a rich repository of longitudinal data on cancer incidence, prevalence, treatment, survival, and patient demographics. Its rigorous data collection and validation protocols make it a globally esteemed resource for epidemiological research and clinical outcomes analysis. For this study, detailed data on patients diagnosed with CVC between 2000 and 2019 were retrieved using the SEER*Stat software (version 8.4.2). The SEER program’s extensive, high-quality datasets enabled a thorough and reliable investigation of CVC epidemiology and outcomes within a large, population-based cohort.

Patients diagnosed with CVC, a rare subtype of cutaneous squamous cell carcinoma, from 2000 to 2019 were identified via ICD-O-3 histology code 8051/3 and primary site codes restricted to skin (C44.0–C44.9). Cases with primary sites corresponding to non-cutaneous locations, including the oral cavity, larynx, penis, vulva, and other mucosal sites, were explicitly excluded to ensure the analytical cohort comprised cutaneous primaries only. Only patients with a first primary malignancy diagnosis of CVC were included in the analysis. Inclusion criteria were age 15–75 years and a microscopically confirmed diagnosis. Exclusion criteria included survival time less than 1 month (22), diagnosis based solely on autopsy or death certificate, unknown race/ethnicity, missing median household income, incomplete demographic or clinical data, unknown primary tumor site, or missing geographic residence information. The study complied with the Declaration of Helsinki ethical principles. Institutional review board approval was waived because SEER data are publicly available and fully anonymized.

To assess the potential upward bias introduced by excluding patients with survival <1 month, a sensitivity analysis was conducted, including these cases. The impact on overall relative survival estimates was minimal, and the primary analysis excluding these patients is presented throughout the manuscript.

### 2.2 Data processing and variable selection

Socioeconomic status (SES), a recognized determinant of health disparities, was incorporated into the analysis in accordance with established guidelines (23, 24). SES indicators comprised median household income and area of residence. Median household income data were derived from the American Community Survey (ACS) as linked to SEER county-level records; these values are time-dependent and updated periodically within SEER to reflect contemporary census estimates. Demographic and clinical variables collected were sex, race/ethnicity, age at diagnosis, median household income, geographic residence, diagnosis date, last follow-up date, and survival status.

Age at diagnosis was categorized into five groups: 15–44, 45–54, 55–64, 65–74, and ≥75 years. Race/ethnicity was classified as White, Black, and Other (including American Indian/Alaska Native, Asian, and Pacific Islander). Median household income as a proxy for SES was stratified into five levels: <$35,000, $35,000–44,999, $45,000–59,999, $60,000–74,999, and ≥$75,000. Diagnosis periods were grouped into four intervals: 2000–2004, 2005–2009, 2010–2014, and 2015–2019.

### 2.3 Statistical analysis

Observed survival rate (OSR) refers to the proportion of patients alive at a given time point regardless of cause of death. Expected survival rate (ESR) is derived from general population life tables matched for age, sex, and calendar year, representing the survival expected in the absence of the cancer of interest. Relative survival rate (RSR) is calculated as the ratio of OSR to ESR, providing a net survival estimate that adjusts for background mortality without requiring cause-of-death information (24). The calculation formula is:

RSR=Sobs/Sexp×100

Where Sobs is the observed survival rate of cancer patients, and Sexp is the expected survival rate of the matched general population. The RSR at time k can be expressed as (25):

$$R_{i}=\frac{\bar{S_{k}}}{S_{k}}$$

Where Ri denotes the relative survival rate at time k, $\bar{S_{k}}$ the observed survival rate, and 𝑆^∗^_𝑘_ the expected survival rate. For the 5-year RSR, *k* = 5.

**Figure 1:**
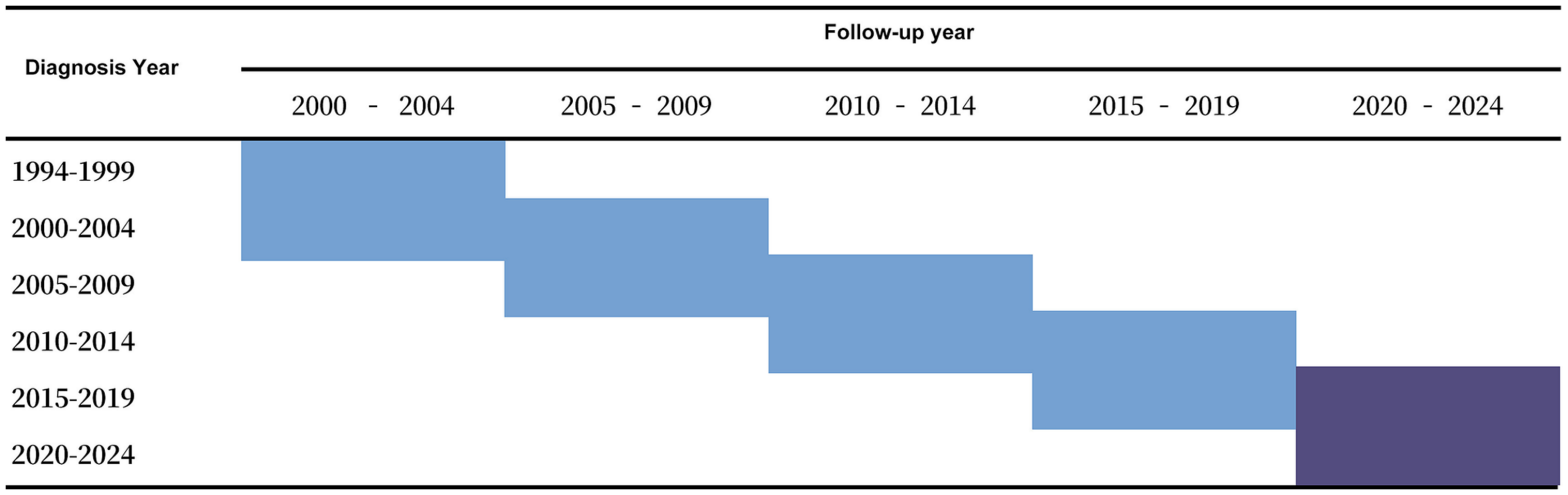
Illustration of the data used to calculate the 5-year relative survival estimates for patients’ diagnosis during the periods 2000–2004, 2005–2009, 2010–2014, and 2015–2019, as well as the predicted estimates for 2020–2024, using period analysis.

Expected survival rates stratified by age and sex were obtained from life tables published by the U.S. Centers for Disease Control and Prevention, covering calendar years 2000–2019, and matched to patients by age, sex, and diagnosis year (26). Observed and expected survival rates were estimated using the Ederer II method (27, 28). RSRs were calculated for four diagnosis intervals (2000–2004, 2005–2009, 2010–2014, and 2015–2019) using the period () function from the Period R package. The data structure underlying this period analysis approach, including the diagnosis and follow-up windows used to calculate RSRs for each interval and the projected 2020–2024 period, is schematically illustrated in Figure 1. Late entry (left truncation) was handled by restricting risk sets to individuals alive and under follow-up within each specified calendar period window, consistent with standard period analysis methodology as described by Brenner and Hakulinen (16).

A generalized linear model (GLM) with a complementary log–log (cloglog) link function was applied to RSR estimates across the four observed diagnosis periods to predict 3- and 5-year RSRs for patients diagnosed between 2020 and 2024. Covariates included diagnosis period as the primary predictor, with subgroup-specific models fitted separately for each stratification variable (sex, age group, race, household income, and residential area). Projected RSRs for 2020–2024 were estimated by extrapolating the fitted GLM trend, with 95% confidence intervals derived from the standard errors of the model coefficients.

Categorical variables were compared using Chi-square tests (χ^2^), and Z-tests were applied to compare survival rates. Trend analyses evaluated temporal changes in survival within groups. All tests were two-sided with significance level *α* = 0.05. *p*-values < 0.05 were considered statistically significant. All subgroup analyses are exploratory in nature. Given the multiplicity of comparisons performed, Z-test results should be interpreted with caution, and findings are hypothesis-generating rather than confirmatory.

## 3 Results

### 3.1 Baseline characteristics

A total of 2,031 patients diagnosed with CVC between 2000 and 2019 were included in the present analysis. Of these, 1,028 patients (50.6%) were male and 1,003 (49.4%) were female, demonstrating an approximately equal distribution by sex. The age distribution of the cohort revealed that the largest proportion of patients were aged 75 years or older (*n* = 761, 37.5%), followed by those aged 65–74 years (*n* = 475, 23.4%) and 55–64 years (*n* = 459, 22.6%). Patients in the 45–54 years age group accounted for 12.1% of the study population (*n* = 246), while those in the youngest category, 15–44 years, comprised 5.3% (*n* = 107). This pattern is consistent with the increased incidence of CVC in older individuals.

The majority of patients resided in metropolitan areas (*n* = 1,747, 86.0%), whereas a smaller proportion were from non-metropolitan (rural and suburban) regions (*n* = 299, 14.7%). Regarding racial and ethnic composition, most patients were White (*n* = 1,764; 86.9%), with Black (*n* = 130, 6.4%) and Other (including American Indian/Alaska Native, Asian, and Pacific Islander; *n* = 137, 6.8%) groups each representing <10% of the population.

Socioeconomic status, as indicated by area-level median household income, showed that 820 patients (40.4%) lived in areas with a median income of $75,000 or higher. An additional 711 patients (35.0%) resided in areas with incomes between $60,000 and $74,999; 394 (19.4%) were in the $45,000–$59,999 range; and 123 (6.1%) lived in areas with median incomes of $35,000–$44,999 or <$35,000. A detailed summary of these baseline characteristics is provided in Table 1.

**Table 1:** Baseline characteristics of patients with cutaneous verrucous carcinoma identified from 2000 to 2019 in the SEER database.

Characteristics	Absolute cases and proportions				X^2^	*p*
	2000–2004 (*n* %)	2005–2009 (*n* %)	2010–2014 (*n* %)	2015–2019 (*n* %)		
Overall	459(23%)	491 (24%)	572 (28%)	509 (25%)		
Sex					13.21	0.004
Male	208 (20%)	267 (26%)	274 (27%)	279 (27%)		
Female	251 (25%)	224 (22%)	298 (30%)	230 (23%)		
Race					6.91	0.33
White	403 (28%)	434 (25%)	485 (21%)	442 (25%)		
Black	32 (25%)	28 (22%)	43 (33%)	27 (21%)		
Others	24 (18%)	29 (21%)	44 (32%)	40 (29%)		
Age at diagnosis					36.60	0.0002
15–44 years	38 (36%)	27 (25%)	20 (19%)	22 (21%)		
45–54 year	50 (20%)	80 (33%)	70 (28%)	46 (19%)		
55–64 year	91 (20%)	116 (25%)	128 (28%)	124 (27%)		
65–74 year	99 (21%)	107 (22%)	128 (27%)	141 (30%)		
75+ year	183 (24%)	163 (21%)	232 (31%)	184 (24%)		
Household income					52.18	<0.01
<$35,000, $35,000–$44,999	23 (19%)	25 (20%)	47 (38%)	28 (23%)		
$45,000–$59,999	62 (16%)	118 (30%)	119 (30%)	95 (24%)		
$60,000–$74,999	152 (22%)	151 (21%)	235 (33%)	173 (24%)		
$75,000+	224 (27%)	199 (24%)	176 (22%)	221 (27%)		
Residential area					4.356	0.226
Metropolitan area	405 (23%)	414 (24%)	484 (28%)	444 (25%)		
Rural area	55 (18%)	78 (26%)	93 (31%)	73 (25%)		

Percentages may not total 100% due to rounding.

### 3.2 Trends in relative survival of CVC by age group

**Figure 2:**
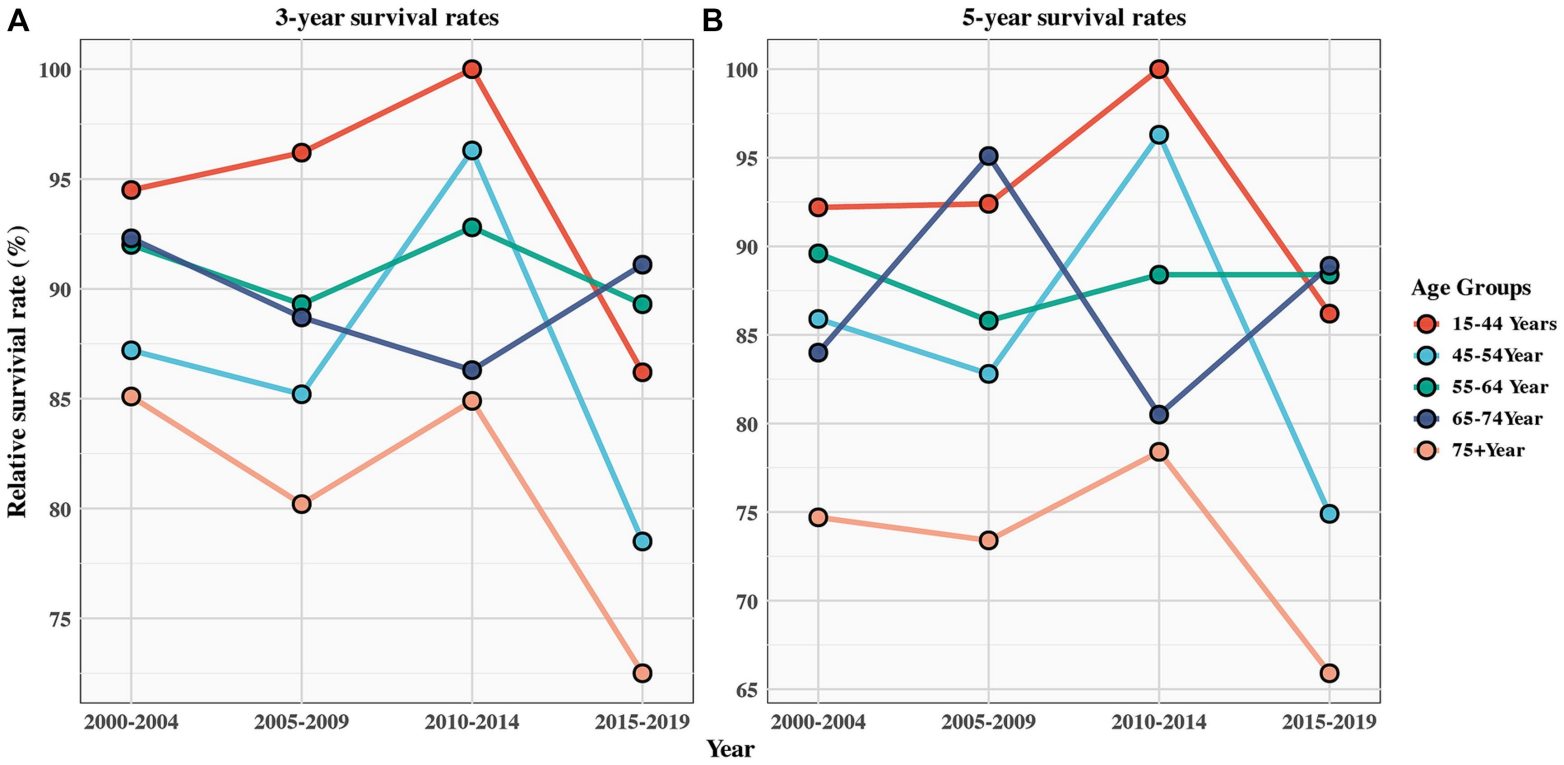
Trends in 3- and 5-year relative survival rates of patients with cutaneous verrucous carcinoma across four time periods: 2000–2004, 2005–2009, 2010–2014, and 2015–2019. **(A)** Three-year relative survival by age group. **(B)** Five-year relative survival by age group.

The analysis of RSRs at 3 and 5 years among patients with CVC across different age groups is summarized in Table 2. When comparing patients diagnosed between 2000 and 2004 and those diagnosed during 2015–2019, a declining trend in both 3- and 5-year RSRs was observed across most age categories, indicating that survival improvements were not uniform across the study period. In each age group, the 3-year RSRs remained higher than the corresponding 5-year RSRs, reflecting the expected decline in survival over extended follow-up. In the most recent period (2015–2019), patients aged 75 years or older had 3- and 5-year RSRs of 72.5% (95% CI: 72.37–72.63%) and 65.9% (95% CI: 65.65–66.15%), respectively, demonstrating the lowest survival among all groups. In contrast, those aged 65–74 years had 3- and 5-year RSRs of 91.1% (95% CI: 91.02–91.18%) and 88.9% (95% CI: 88.78–89.02%), and the 55–64 year group showed rates of 89.3% (95% CI: 89.23–89.37%) and 88.4% (95% CI: 88.31–88.49%). For the 45–54-year group, the respective RSRs were 78.5% (95% CI: 78.41–78.59%) and 74.9% (95% CI: 74.77–75.03%), while the youngest cohort (15–44 years) achieved equivalent 3- and 5-year RSRs of 86.2% (95% CI: 86.01–86.39%). Figure 2 visually illustrates these patterns. Trend analysis over the full study period revealed a downward trajectory in RSRs for all age groups, with older patients consistently demonstrating poorer outcomes. These findings are exploratory and should be interpreted in light of the multiplicity of subgroup comparisons performed. Generalized linear model projections for the years 2020–2024 anticipate that the highest future RSRs will occur in the 15–44-year group (93.3% at 3 years and 93.2% at 5 years), whereas the lowest rates are projected for patients aged 75 years and older (74.0 and 67.0%, respectively). These findings indicate an age-dependent disparity in survival outcomes, with older patients exhibiting consistently poorer relative survival following CVC diagnosis.

**Table 2:** Three- and five-year relative survival rates of cutaneous verrucous carcinoma (CVC) patients by age group (2000–2019), and projected rates for survival rates (2020–2024).

Age groups (year)	2000–2004	2005–2009	2010–2014	2015–2019	2020–2024
3-year survival rates					
15–44 years	94.5 ± 0.039	96.2 ± 0.043	100 ± 0.000	86.2 ± 0.096	93.318
45–54 year	87.2 ± 0.048	85.2 ± 0.045	96.3 ± 0.029	78.5 ± 0.045	82.285
55–64 year	92.0 ± 0.034	89.3 ± 0.035	92.8 ± 0.029	89.3 ± 0.038	90.180
65–74 year	92.3 ± 0.037	88.7 ± 0.044	86.3 ± 0.043	91.1 ± 0.043	89.095
75+ year	85.1 ± 0.050	80.2 ± 0.056	84.9 ± 0.046	72.5 ± 0.064	73.960
5-year survival rates					
15–44 years	92.2 ± 0.048	92.4 ± 0.059	100 ± 0.000	86.2 ± 0.096	93.169
45–54 year	85.9 ± 0.050	82.8 ± 0.049	96.3 ± 0.029	74.9 ± 0.067	80.555
55–64 year	89.6 ± 0.040	85.8 ± 0.042	88.4 ± 0.046	88.4 ± 0.046	87.401
65–74 year	84.0 ± 0.051	95.1 ± 0.026	80.5 ± 0.052	88.9 ± 0.059	86.634
75+ year	74.7 ± 0.065	73.4 ± 0.069	78.4 ± 0.067	65.9 ± 0.130	67.016

Data are means ± standard error of the means.

### 3.3 Gender differences in 3- and 5-year relative survival rates

**Figure 3:**
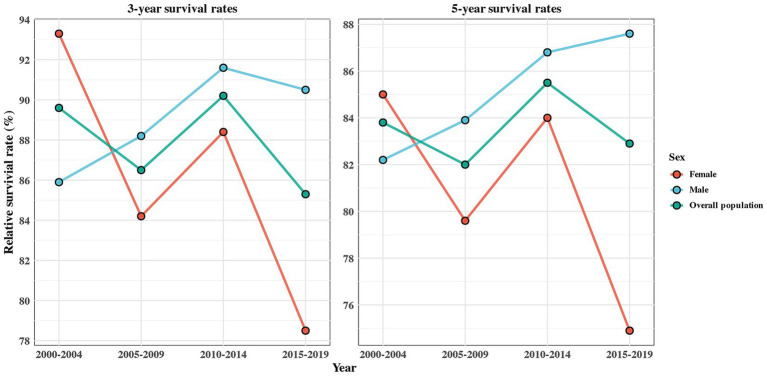
Trends in 3- and 5-year relative survival rates of patients with cutaneous verrucous carcinoma by sex, across the periods 2000–2004, 2005–2009, 2010–2014, and 2015–2019.

Analysis of sex differences in 3- and 5-year RSRs for CVC revealed diverging trends between males and females over the study period. As detailed in Table 3, overall 3- and 5-year RSRs declined in the total population and among females from 2000–2004 to 2015–2019, reaching 85.3% (95% CI: 85.25–85.35%) and 82.9% (95% CI: 82.83–82.97%) for the entire cohort, and 78.5% (95% CI: 78.41–78.59%) and 74.9% (95% CI: 74.77–75.03%) among females, respectively. In contrast, males demonstrated a rising trend, with 3- and 5-year RSRs of 90.5% (95% CI: 90.44–90.56%) and 87.6% (95% CI: 87.52–87.68%) in the 2015–2019 period. These contrasting survival trajectories are depicted in Figure 3.

**Table 3:** Three- and five-year relative survival rates of patients with cutaneous verrucous carcinoma by sex (2000–2019), and projected rates for 2020–2024 (x ± SE, %).

Sex	2000–2004	2005–2009	2010–2014	2015–2019	2020–2024
3-year survival rates					
Overall population	89.6 ± 0.022	86.5 ± 0.023	90.2 ± 0.021	85.3 ± 0.027	86.823
Male	85.9 ± 0.030	88.2 ± 0.028	91.6 ± 0.026	90.5 ± 0.032	93.335
Female	93.3 ± 0.031	84.2 ± 0.036	88.4 ± 0.035	78.5 ± 0.045	78.337
5-year survival rates					
Overall population	83.8 ± 0.027	82.0 ± 0.027	85.5 ± 0.026	82.9 ± 0.037	82.526
Male	82.2 ± 0.036	83.9 ± 0.033	86.8 ± 0.033	87.6 ± 0.043	89.221
Female	85.0 ± 0.041	79.6 ± 0.043	84.0 ± 0.043	74.9 ± 0.067	73.583

Survival rates represent relative survival rates; data are presented as means ± standard errors.

Projections using generalized linear modeling suggest that this divergence will persist into 2020–2024. Males are expected to achieve further improvement, with predicted 3- and 5-year RSRs of 93.3 and 89.2%, respectively. Conversely, females are projected to experience additional declines, with 3- and 5-year RSRs estimated at 78.3 and 73.6%. These diverging trends between sexes may reflect differences in tumor site distribution, sex-specific tumor biology, including genetic and epigenetic differences in carcinogenesis, healthcare-seeking behavior, or hormonal influences on immune response, and warrant further investigation. This persistent and widening survival gap between sexes contrasts with some previous literature; several studies have reported either higher or comparable survival in females, while others have noted male predominance in incidence and mortality. These subgroup findings are exploratory and should be interpreted with appropriate caution, given the multiplicity of comparisons made.

### 3.4 Racial disparities in 3- and 5-year relative survival rates

**Figure 4:**
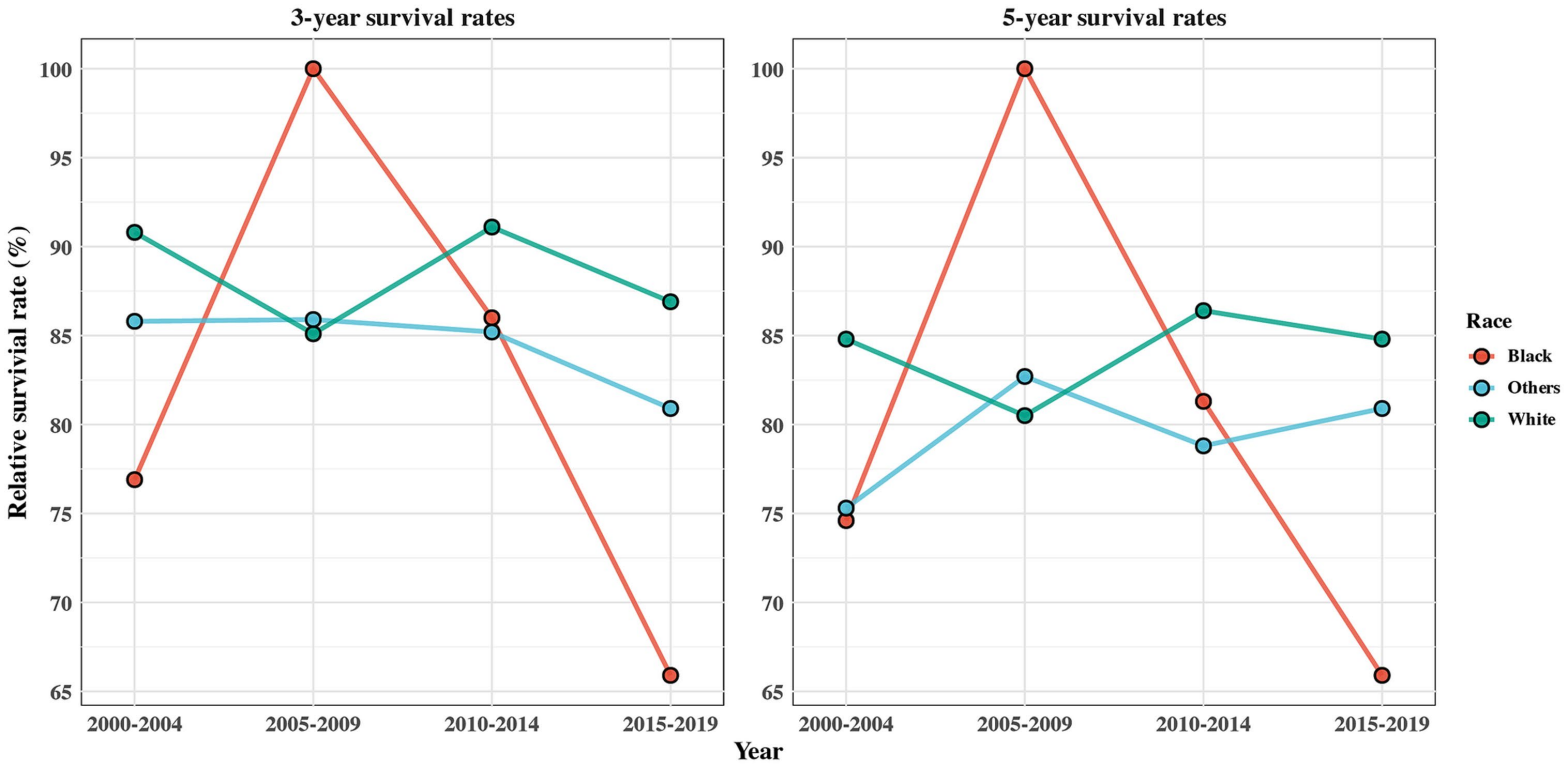
Trends in 3- and 5-year relative survival rates of patients with cutaneous verrucous carcinoma by race across the periods 2000–2004, 2005–2009, 2010–2014, and 2015–2019.

Analysis of racial disparities in 3- and 5-year RSRs among patients with CVC reveals persistent and significant differences across ethnic groups. As detailed in Table 4, during the 2015–2019 period, White patients exhibited the highest survival rates, with 3- and 5-year RSRs of 86.9% (95% CI: 86.84–86.96%) and 84.8% (95% CI: 84.74–84.86%), respectively. In contrast, Black patients reported markedly lower survival, with both 3- and 5-year RSRs at 65.9% (95% CI: 65.63–66.17%). Statistical comparisons across all time periods demonstrated that White and other ethnic groups consistently had significantly higher survival rates compared to Black patients (*p* < 0.05), a trend further illustrated in Figure 4.

**Table 4:** Three- and five-year relative survival rates of patients with cutaneous verrucous carcinoma by race (2000–2019), and projected rates for 2020–2024 (x ± SE, %).

Race	2000–2004	2005–2009	2010–2014	2015–2019	2020–2024
3-year survival rates					
White	90.8 ± 0.023	85.1 ± 0.024	91.1 ± 0.023	86.9 ± 0.028	88.457
Black	76.9 ± 0.088	100 ± 0.000	86.0 ± 0.079	65.9 ± 0.138	71.302
Others	85.8 ± 0.096	85.9 ± 0.096	85.2 ± 0.065	80.9 ± 0.084	84.077
5-year survival rates					
White	84.8 ± 0.029	80.5 ± 0.029	86.4 ± 0.029	84.8 ± 0.041	84.108
Black	74.6 ± 0.093	100 ± 0.000	81.3 ± 0.087	65.9 ± 0.138	69.784
Others	75.3 ± 11.8	82.7 ± 0.107	78.8 ± 0.079	80.9 ± 0.084	79.074

Survival rates represent relative survival rates; data are presented as means ± standard errors.

Projections using a generalized linear model for the 2020–2024 period indicate the continuation of these disparities. White patients are anticipated to maintain the highest 3- and 5-year RSRs at 88.46 and 84.11%. In comparison, Black patients are projected to have the lowest survival, with 3- and 5-year RSRs of 71.30 and 69.78%. Other ethnicities are expected to have intermediate rates, with 3- and 5-year RSRs of 84.08 and 79.07%, respectively. These findings are consistent with broader research on skin cancer outcomes, which has demonstrated that Black patients, as well as other minority groups such as American Indian/Alaska Native and Asian or Pacific Islanders, experience significantly lower survival rates than White patients.

Multiple factors contribute to these racial disparities, including delayed disease detection, differences in tumor biology, limited access to healthcare resources, higher rates of comorbidities, and socioeconomic barriers. Notably, skin cancers in patients with darker skin types often present in sun-protected or atypical locations, which may contribute to delayed diagnosis and more advanced disease at the time of presentation. Lower awareness of skin cancer risk in these populations further exacerbates this challenge. Collectively, these disparities underscore the urgent need for improved access to dermatologic care, enhanced public awareness, and targeted screening efforts in underserved minority groups.

### 3.5 Household income and relative survival rates

**Figure 5:**
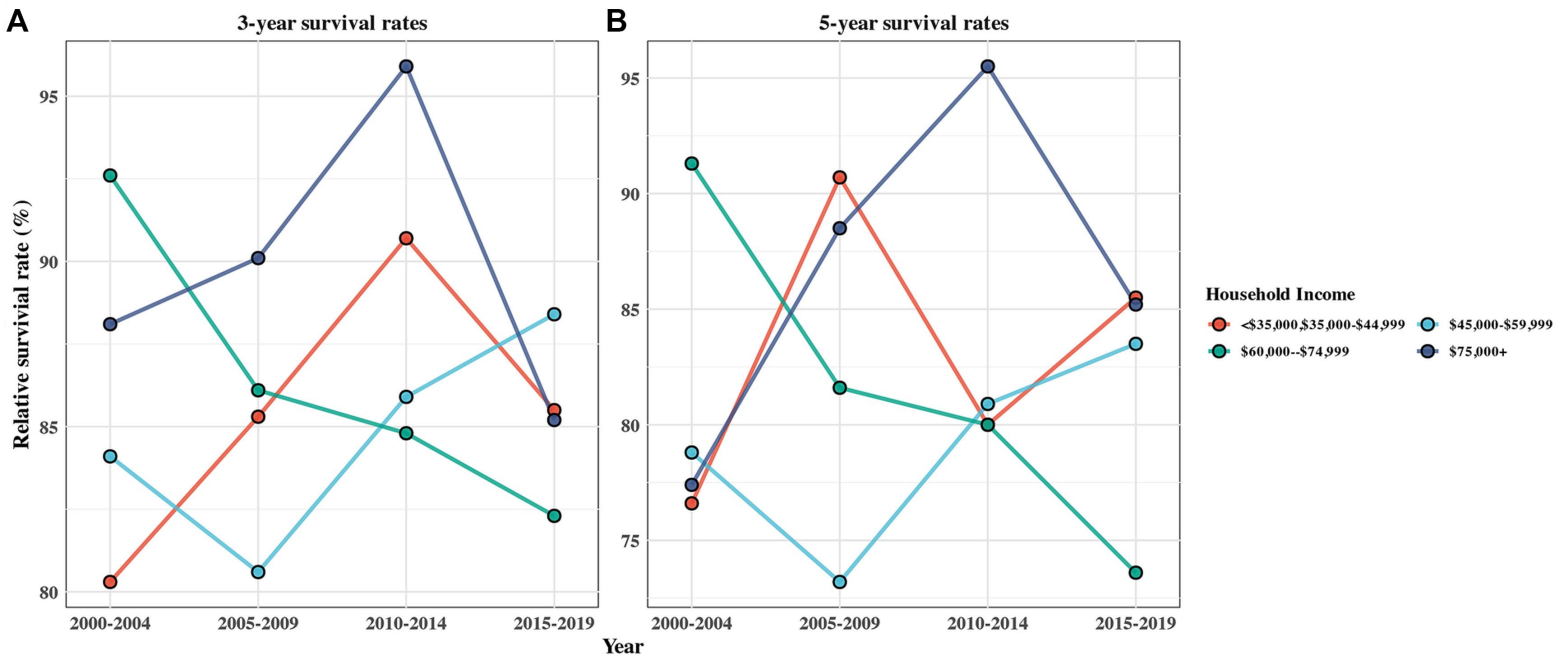
Trends in three- and five-year relative survival rates of patients with cutaneous verrucous carcinoma, stratified by household income across four time periods: 2000–2004, 2005–2009, 2010–2014, and 2015–2019. **(A)** Three-year relative survival rate by household income group. **(B)** Five-year relative survival rate by household income group.

The 3- and 5-year relative survival rates of patients with CVC, stratified by household income levels, are presented in Table 5. In the period 2015–2019, patients with household incomes between $45,000 and $59,999 had the highest 3- relative survival rate at 88.4% (95% CI: 88.29–88.51%). The highest 5-year relative survival rate of 85.5% (95% CI: 85.36–85.64%) was observed in patients with incomes below $35,000 and those with incomes between $35,000 and $44,999. This finding should be interpreted with caution given the relatively small sample size in the lowest income category (*n* = 123), which may have contributed to unstable estimates. Patients with incomes between $60,000 and $74,999 showed the lowest survival outcomes in the most recent period, with 3- and 5-year relative survival rates of 82.3% (95% CI: 82.21–82.39%) and 73.6% (95% CI: 73.46–73.74%), respectively. The survival trends across different income groups are illustrated in Figure 5.

**Table 5:** Three- and five-year relative survival rates of patients with cutaneous verrucous carcinoma by household income (2000–2019), with projected rates for 2020–2024 (x ± SE, %).

Household income	2000–2004	2005–2009	2010–2014	2015–2019	2020–2024
3-year survival rates					
<$35,000, $35,000–$44,999	80.3 ± 0.072	85.3 ± 0.066	90.7 ± 0.052	85.5 ± 0.072	89.707
$45,000–$59,999	84.1 ± 0.060	80.6 ± 0.047	85.9 ± 0.052	88.4 ± 0.057	88.835
$60,000–$74,999	92.6 ± 0.031	86.1 ± 0.04	84.8 ± 0.036	82.3 ± 0.047	76.605
$75,000+	88.1 ± 0.033	90.1 ± 0.034	95.9 ± 0.03	85.2 ± 0.038	92.632
5-year survival rates					
<$35,000, $35,000–$44,999	76.6 ± 0.092	90.7 ± 0.052	80.0 ± 0.068	85.5 ± 0.072	87.345
$45,000–$59,999	78.8 ± 0.072	73.2 ± 0.055	80.9 ± 0.062	83.5 ± 0.059	87.210
$60,000–$74,999	91.3 ± 0.040	81.6 ± 0.048	80.0 ± 0.042	73.6 ± 0.071	72.329
$75,000+	77.4 ± 0.042	88.5 ± 0.038	95.5 ± 0.039	85.2 ± 0.038	89.358

Survival rates represent relative survival rates; data are presented as means ± standard errors.

According to trend projections based on generalized linear modeling, during the forecast period 2020–2024, patients with household incomes above $75,000 are expected to have the highest relative survival rates, reaching 92.6% at 3 years and 89.4% at 5 years. The lowest predicted survival rates were identified in the $60,000–$74,999 income group (76.6% at 3 years and 72.3% at 5 years). For patients with incomes below $35,000, between $35,000–$44,999, and $45,000–$59,999, the projected 3- and 5-year relative survival rates were 89.7 and 87.3%, and 88.8 and 87.2%, respectively. These subgroup findings are exploratory in nature and should be interpreted with caution.

### 3.6 Trends in relative survival of patients with CVC by residential area

**Figure 6:**
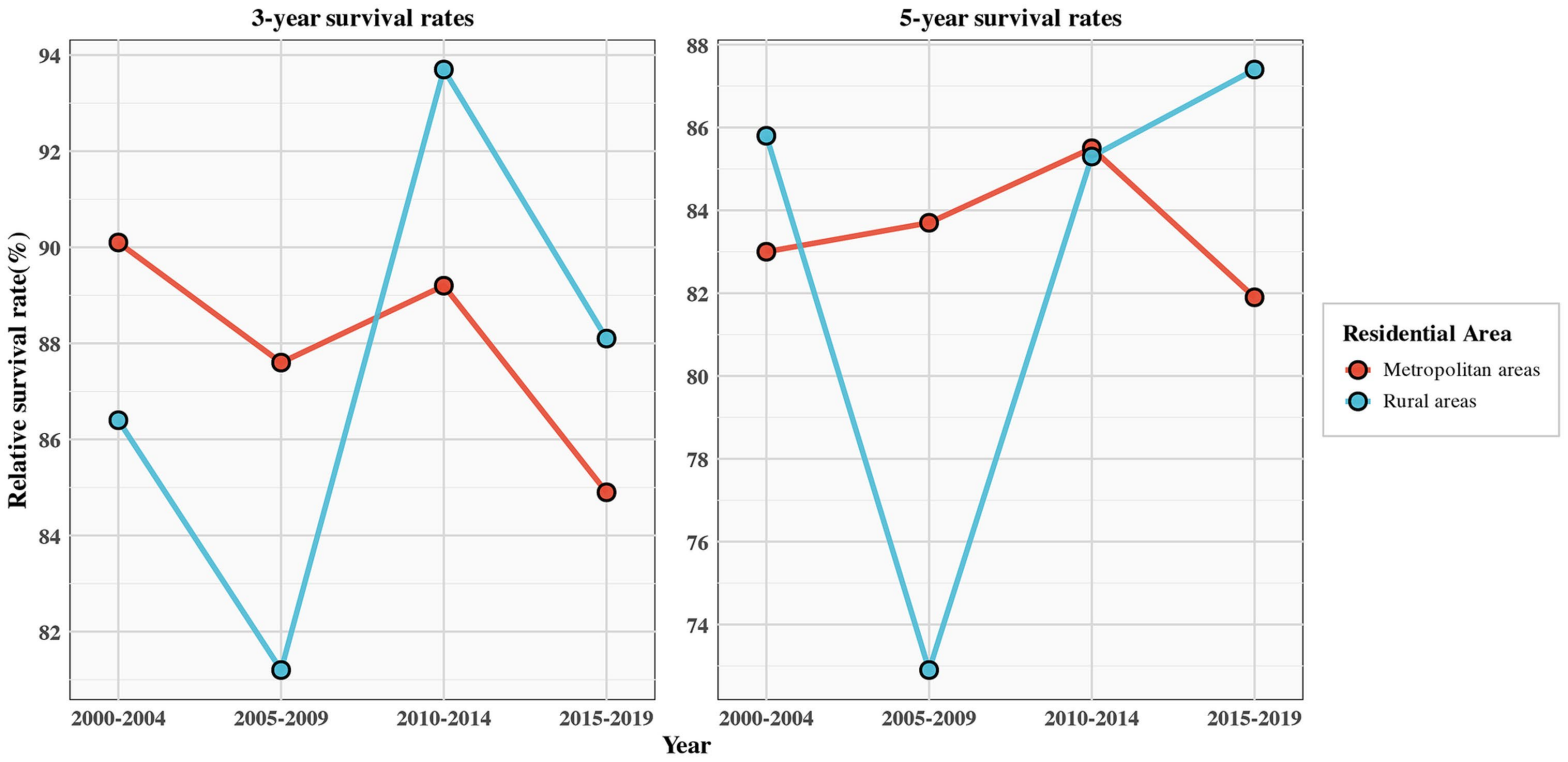
Trends in 3- and 5-year relative survival rates of patients with cutaneous verrucous carcinoma by residential (metropolitan vs. rural) across the periods 2000–2004, 2005–2009, 2010–2014, and 2015–2019.

The relative survival rates among patients with CVC exhibited notable differences based on residential location, as summarized in Table 6. Period analysis revealed a significant improvement in survival outcomes for patients residing in rural areas over the study interval. During 2015–2019, the 3- and 5-year relative survival rates in rural regions reached 88.1% (95% CI: 87.97–88.23%) and 87.4% (95% CI: 87.24–87.56%), respectively, surpassing those observed in urban counterparts, where the 3- and 5-year rates were 84.9% (95% CI: 84.84–84.96%) and 81.9% (95% CI: 81.82–81.98%), respectively. These patterns are further visualized in Figure 6.

**Table 6:** Three- and five-year relative survival rates of patients with cutaneous verrucous carcinoma by residential area (2000–2019), with projected rates for 2020–2024 (x ± SE, %).

Residential area	2000–2004	2005–2009	2010–2014	2015–2019	2020–2024
3-year survival rates					
Metropolitan areas	90.1 ± 0.024	87.6 ± 0.024	89.2 ± 0.024	84.9 ± 0.029	86.027
Rural areas	86.4 ± 0.055	81.2 ± 0.058	93.7 ± 0.041	88.1 ± 0.064	91.543
5-year survival rates					
Metropolitan areas	83.0 ± 0.03	83.7 ± 0.029	85.5 ± 0.029	81.9 ± 0.041	81.698
Rural areas	85.8 ± 0.065	72.9 ± 0.067	85.3 ± 0.066	87.4 ± 0.084	86.827

Survival rates represent relative survival rates; data are presented as means ± standard errors.

Projections using a generalized linear model for the 2020–2024 period suggest that this favorable survival trend for rural populations will persist. Patients living in rural areas are expected to achieve the highest relative survival rates, with predicted 3- and 5-year rates of 91.5 and 86.8%, respectively. In contrast, those in metropolitan areas are projected to have the lowest survival outcomes, at 86.0% (3-year) and 81.7% (5-year). These findings may reflect differences in population demographics, patterns of healthcare utilization, stage at presentation, or disparities in access to specialized dermatologic and oncologic services. These subgroup findings are exploratory and require cautious interpretation.

### 3.7 Comparative relative survival rates with other cutaneous malignancies

CVC survival (82.9%) is comparable to or slightly superior to conventional cutaneous SCC (80–85%), the parent category of which CVC is a rare well-differentiated subtype. CVC demonstrates substantially superior 5-year survival compared to melanoma overall (72–73%), though melanoma at Stage I presents with superior outcomes (95%). CVC substantially trails BCC (>95%), reflecting fundamental differences in tumor biology.

## 4 Discussion

Non-melanoma skin cancers, including cSCC and its rare variants such as CVC, have emerged as a significant global health burden in recent decades (29). CVC, a rare and well-differentiated cSCC subtype, is characterized by indolent growth, low metastatic potential, and a deceptively benign appearance, often resulting in diagnostic delays or misdiagnosis (30). According to the Global Burden of Disease (GBD) 2017 study (4), the global incidence and prevalence of cSCC, the parent category of CVC, rose by 310% from 1990 to 2017, the highest increase among all cutaneous malignancies. Despite this rising burden, CVC remains underreported as a distinct entity, and survival-focused studies are limited, underscoring the need for comprehensive prognostic analyses to inform clinical decision-making.

To our knowledge, this is the first study to evaluate relative survival rates (RSRs) for CVC across four consecutive 5-year intervals (2000–2019) and to project survival trends through 2024. Survival trends varied across subgroups. While the overall 5-year RSR in the most recent period (2015–2019) was 82.9%, reflecting broadly stable outcomes at the population level, subgroup analyses revealed declining RSRs across age groups, with older patients showing significantly poorer survival. Model-based projections suggest modest improvement through 2024, driven primarily by gains in younger age groups. These findings align with previous studies identifying age as an independent prognostic factor in CVC (31, 32). The lower survival in older adults may be attributable to reduced treatment tolerance (33), delayed diagnosis due to slow tumor progression (34), higher comorbidity burden, and limited access to specialized care (35). Conversely, younger patients more frequently present with localized, low-stage, and low-grade tumors, contributing to better prognoses (36–38).

Interestingly, sex-based disparities were observed. While male patients demonstrated improved RSRs over time, female patients exhibited a declining trend, contrary to earlier reports suggesting stable or superior survival among women (39). This divergence may reflect sex-specific tumor biology, including genetic and epigenetic differences in response to carcinogenesis (40, 41). For instance, disparities in somatic mutations such as BAP1 and *β*-catenin may influence tumor behavior and treatment responsiveness (42). Additionally, hormonal influences, differences in UV exposure patterns by sex, and sex-specific immune responses may further contribute to these diverging survival trajectories. These sex-based findings are exploratory in nature and should be interpreted with appropriate caution given the multiplicity of subgroup comparisons performed.

Furthermore, our analysis revealed heterogeneous trends in RSRs across subgroups from 2000–2004 to 2015–2019. While male patients and some income groups demonstrated improving survival, overall population-level RSRs remained broadly stable, with declining trends observed in females, older age groups, and certain socioeconomic strata. These findings highlight the importance of disaggregated survival analyses rather than reliance on aggregate trends alone. Racial disparities were evident, with White patients consistently exhibiting higher survival than Black and other racial groups. This discrepancy may be partly explained by socioeconomic differences, as White patients are more likely to benefit from early detection and timely treatment due to better healthcare access (43). In contrast, structural barriers such as medical mistrust, lack of awareness, limited access to dermatologic care, disparities in insurance coverage, and physician bias may contribute to poorer outcomes among Black patients (44–46).

Household income also played a critical role in survival. Between 2000 and 2019, patients with annual income below $45,000 experienced gradual improvements in both 3- and 5-year RSRs, likely due to expanded policy coverage and improvements in healthcare infrastructure (47, 48). However, projections for 2020–2024 suggest that only patients earning above $75,000 will continue to experience RSR gains. This emerging disparity likely reflects uneven distribution of healthcare resources, insurance coverage gaps, regional differences in care quality, and behavioral risk factors more prevalent among socioeconomically disadvantaged populations (48).

Geographic disparities in survival were notable and somewhat counterintuitive. Contrary to the usual epidemiological pattern in which rural populations experience worse outcomes due to limited healthcare access, our analysis showed higher five-year relative survival in rural compared with metropolitan CVC patients during 2015–2019. This observation requires careful interpretation. Several factors may contribute to this unexpected rural advantage. CVC often arises in non-sun-exposed but easily examinable areas, allowing similar opportunities for self-detection across settings, and its indolent growth may lessen the impact of delayed care. Differences in population composition may also play a role; rural patients may differ from urban patients in age structure or comorbidity burden, which could influence survival independently of access. Variation in health-seeking behavior for visible lesions may further reduce barriers to early evaluation in rural settings. In contrast, urban populations may carry a higher burden of multimorbidity and psychosocial stressors, which can adversely affect overall survival and treatment adherence. Additionally, differences in registry follow-up and data completeness between rural and urban areas may influence survival estimates. These findings should be interpreted cautiously. The observed rural survival advantage is exploratory and hypothesis-generating, and the underlying mechanisms remain unclear. Further studies incorporating stage at diagnosis, treatment patterns, comorbidity profiles, and demographic stratification are needed to validate and explain this pattern (48, 49). Urban lifestyle factors such as social isolation, depression, and reduced health-seeking behaviors may further exacerbate poor outcomes (50, 51). In contrast, despite challenges in cancer registry coverage and data quality (52), targeted improvements in regional healthcare delivery may have contributed to survival gains among rural patients.

Health disparities in CVC survival are multifactorial, rooted in systemic inequities involving education, employment, healthcare access, insurance, and social justice. Individuals from disadvantaged backgrounds often encounter significant barriers to timely and effective care, resulting in delayed diagnoses and inferior outcomes (50, 53). Addressing these disparities requires comprehensive healthcare system reforms, including equitable access to care, targeted policy interventions, and increased investment in public health infrastructure.

Collaborative efforts with public health agencies are crucial to enhancing early detection programs, raising disease awareness, and improving access to timely and effective treatment. Such initiatives are vital to reducing CVC-related mortality and narrowing survival gaps, particularly in vulnerable populations (54). Additionally, educational programs targeting both healthcare providers and the general public should emphasize prevention and early diagnosis, given the high curability of skin cancers at early stages (55).

This study has several limitations. First, the data were derived exclusively from the US-based SEER database, which may limit generalizability to other populations. Second, the lack of treatment-specific data (e.g., surgical approach, radiotherapy, chemotherapy) precludes analysis of the impact of specific therapies on survival. Third, potential misclassification or underreporting, particularly of early-stage or asymptomatic cases, may lead to underestimation of the true incidence. Fourth, as a retrospective study, our findings are subject to selection bias, confounding, and inherent limitations of registry-based data, including variability in data quality and coding across regions and time. Fifth, the exclusion of patients with survival in <1 month, while reducing immortal time bias, may introduce a degree of upward bias in RS estimates; however, sensitivity analyses confirmed minimal impact on overall findings. Finally, the multiplicity of subgroup comparisons performed in this study increases the risk of type I error; all subgroup findings should therefore be considered hypothesis-generating and interpreted with appropriate caution. An important limitation of this study relates to the accuracy of cutaneous verrucous carcinoma (CVC) identification and classification within the SEER database. Unlike melanoma and other major malignancies, cutaneous squamous cell carcinoma (SCC) and its rare variants, including CVC, are not consistently captured as distinct reportable entities across all SEER registries. As a result, CVC cases may be miscoded or subsumed under broader diagnostic categories such as well-differentiated SCC or SCC not otherwise specified (NOS), leading to potential misclassification. This limitation may contribute to underestimation of the true incidence if genuine CVC cases are recorded as conventional SCC or if the specific morphology code (ICD-O-3: 8051/3) is not accurately assigned, while conversely introducing misclassification bias if non-CVC well-differentiated SCCs are incorrectly coded as CVC, thereby inflating case counts. Furthermore, variability in coding practices and data quality standards across the 17 SEER registries may introduce systematic bias in survival estimates, particularly for rare tumor subtypes such as CVC. To date, no validation studies have specifically evaluated the accuracy of CVC case identification using SEER coding, highlighting the need for future prospective investigations that integrate SEER data with original pathology reports to better quantify and address potential misclassification. A major limitation of this study is the absence of detailed treatment information (surgical approach, margin status, radiotherapy, and chemotherapy) and molecular data (BAP1 and *β*-catenin mutations) in the SEER database. This restricts the evaluation of treatment-specific survival outcomes and the prognostic impact of genetic alterations. The lack of genomic data also limits risk stratification and personalized treatment planning. Future studies integrating comprehensive treatment and molecular profiling are needed to improve the clinical applicability of CVC survival analyses.

## 5 Conclusion

This population-based study evaluated long-term relative survival trends among CVC patients from 2000 to 2019 using period analysis of SEER data. Survival trends were heterogeneous across subgroups, with male patients demonstrating improving RSRs over time while declining trends were observed in females, older age groups, and urban residents. Model-based projections suggest that these diverging patterns are likely to persist through 2020–2024, with continued improvement anticipated primarily in younger and male patients. Persistent racial and socioeconomic disparities, particularly impacting Black individuals and those with limited healthcare access, remain a significant concern. Although overall population-level survival remained broadly stable across the study period, the magnitude of disparities by sex, race, income, and residential area underscores the urgent need for targeted clinical strategies and equity-focused policies to address these gaps and improve outcomes in vulnerable populations.

## Data Availability

The datasets generated and/or analyzed during the current study are available within the article and its supplementary materials. Any further inquiries regarding the data or additional information can be directed to the corresponding authors.
